# Tooth whitening with hydrogen peroxide in adolescents: study protocol for a randomized controlled trial

**DOI:** 10.1186/1745-6215-15-395

**Published:** 2014-10-14

**Authors:** Marcelo Mendes Pinto, Camila Haddad Leal de Godoy, Carolina Carvalho Bortoletto, Silvia Regina Garcia Olivan, Lara Jansiski Motta, Olga Maria Altavista, Katia Lumi, Ana Paula Taboada Sobral, Sandra Kalil Bussadori

**Affiliations:** Children’s Dentistry Sector, Universidade Nove de Julho (UNINOVE), R. Vergueiro, 235 – Liberdade, São Paulo, SP CEP 01504-001 Brazil; Universidade Nove de Julho (UNINOVE), R. Vergueiro, 235 – Liberdade, São Paulo, SP CEP 01504-001 Brazil

**Keywords:** Tooth whitening, Adolescent, Clinical trial

## Abstract

**Background:**

Technological innovations in dental materials have been fueled by the desire of patients to improve the esthetics of their teeth. This emphasis on esthetics has led dentists to seek resources that respect the standards established by society, but without compromising the integrity of the teeth.

**Methods/design:**

The aim of the proposed controlled clinical trial will be to assess colorimetric changes and increased dental sensitivity in adolescent patients submitted to tooth whitening with 6% and 7.5% hydrogen peroxide using home kits with whitening strips. Adolescents aged 12 to 20 years will be allocated to different groups based on treatment (*n* = 16 per group): (1) placebo; (2) 6.0% hydrogen peroxide (White Class with Calcium, FGM); (3) 7.5% hydrogen peroxide (White Class with Calcium, FGM); and (4) 7.5% hydrogen peroxide (Oral B 3D White, Oral-B). After the whitening procedures, the participants will be evaluated using a visual analog scale for tooth sensitivity and digital spectrophotometry to measure changes in color. Descriptive analysis of the data will be performed. Either the chi-squared test or Fisher’s exact test will be used for the determination of associations among the categorical variables. Student’s *t*-test and analysis of variance will be used to compare mean colorimetric data. Pearson’s correlation coefficients will be calculated to determine the strength of correlations among the continuous variables.

**Discussion:**

This randomized trial will provide an opportunity to evaluate products such as whitening strips in comparison to other self-administered methods, especially in adolescents.

**Trial registration:**

The protocol for this study was submitted to Clinical Trials in November 2013 with registration number NCT01998386.

## Background

Technological innovations in dental materials have been fueled by the desire of patients to improve the esthetics of their teeth, which many consider to be an important aspect of quality of life [1, 2]. This emphasis on esthetics has led dentists to seek resources that respect the standards established by society, but without compromising the integrity of the teeth, as the treatment philosophy should be the maximum preservation of sound dental tissues [3, 4].

Tooth discoloration is classified based on the etiology. Extrinsic factors cause superficial changes in color, such as staining due to the consumption of coffee, tea, soft drinks, etc. Intrinsic factors, whether congenital or acquired, cause more complicated discoloration that is difficult to treat, as such discoloration is incorporated into the structure of the teeth and generally only removed through the use of more aggressive procedures that can have a negative effect on sound dental tissues [3, 5, 6].

Esthetic problems in childhood and adolescence can exert a significant influence on one’s psychosocial development and interactions with peers. Shulman *et al*. [7] evaluated 2,495 patients and found that 32% of the children were dissatisfied with the color of their teeth and 19% of the parents shared this dissatisfaction. Indeed, children, adolescents and young adults have become increasingly concerned with dental esthetics, which may have an important psychological impact on quality of life and may be one of the driving forces behind the current demand for dental materials and noninvasive procedures aimed at improving tooth color [8].

Tooth whitening is the procedure most commonly employed by professionals and patients and is considered the least invasive esthetic treatment for improving the appearance of one’s smile. The procedure is simple and inexpensive. However, the expected whitening outcome cannot be guaranteed [1–3].

Whitening can be performed in the dentist’s office or with the use of a home kit [3]. At-home whitening should be supervised by a dental professional and consists of the application of different concentrations of a peroxide gel (carbamide or hydrogen) to the teeth with the aid of personalized molds [9].

The desires of consumers, fueled to some extent by the standards of beauty dictated by society [1], have stimulated the development of over-the-counter products sold in pharmacies and supermarkets without the need for a prescription or professional supervision [1, 10, 11]. These whitening products first appeared in the USA in 2000 as an alternative to dental staining treatment, with a lower cost in comparison to traditional treatment by dentists [10]. In the last 10 years, other over-the-counter whitening agents have been added, such as toothpastes, mouthwashes and chewing gum [10].

Whitening strips and varnishes containing peroxide fall into this category of freely commercialized products without professional supervision, exposing individuals to allergic reactions, the ingestion of the whitening agent and dental sensitivity [12, 13]. Moreover, the lack of professional monitoring indicates there may be a greater risk of the improper use of these products [11].

The safety, efficacy, long-term color stability and discomfort (dental sensitivity and gingival irritation) of over-the-counter whitening products need to be evaluated, quantified and described in the scientific literature. Thus, long-term controlled clinical trials are needed to evaluate products such as whitening strips in comparison to other self-administered methods, especially for adolescents.

The aim of the proposed study is to broaden knowledge of the clinical use and characteristics of self-administered hydrogen-peroxide-based whitening materials for professional use and over-the-counter sales employed and indicated for adolescents aged 12 to 20 years.

### Specific objectives

The specific objectives are to:

- Evaluate and quantify colorimetric changes in young permanent anterior teeth
- Compare the efficiency and efficacy of three gels used for self-administered home whitening and an over-the-counter whitening strip
- Analyze the increase in dental sensitivity for adolescent patients undergoing home whitening with 6% and 7.5% hydrogen peroxide with and without the addition of calcium (whitening strips)
- Assess sensitivity, comfort and acceptance of whitening strips by patients
- Assess patient satisfaction with the method and material used for home whitening

## Materials/design

The present study will be conducted in compliance with the guidelines stipulated for research involving human subjects. It has received approval from the Human Ethics Committee of University Nove de Julho (Brazil), under process number 410.582. Legal guardians will receive information on the objectives and procedures and those agreeing to the participation of their sons and daughters will sign a statement of informed consent.

### Participants

Male and female adolescents enrolled at the Dental Clinic of University Nove de Julho (Sao Paulo, Brazil) will be recruited.

#### Inclusion criteria

Participants will be aged 12 to 20 years, with a diagnosis of altered color on maxillary and mandibular anterior teeth with initial color equivalent to A2 on the Vita scale (Vita Zahnfabrik) and will have a signed statement of informed consent.

#### Exclusion criteria

Participants will be excluded if: they have any dental anomalies (malformation, carious lesions, fractures); they have at least four maxillary and/or mandibular anterior teeth; they have had a known adverse reaction to peroxide; they have used whitening agents (administered in a dental office or at home) in the previous year; they are currently undergoing orthodontic treatment, orthopedic treatment of the jaws or psychological treatment; or they are using medication that can alter the color of the teeth, such as ferrous sulfate.

### Discomfort or expected risk

The volunteers may experience discomfort during the casting of the dental arches and sensitivity after use of the whitening agents. This sensitivity will diminish after the cessation of treatment.

### Information and clarification

The volunteers will receive answers to any questions they may have and clarification regarding issues related to the procedures, risks, benefits and other subjects related to the study. The researchers will commit to providing updated information obtained throughout the study, even if this information could affect the volunteers’ willingness to continue participating. All volunteers will be treated at the dental clinic of the University Nove de Julho to resolve any treatment needs. The participants will also benefit esthetically from the whitening treatment.

### Procedures

The selected participants will be randomly allocated by lots to the different groups, as shown in Table 1.

**Table 1:** **Summary of experimental conditions**

Group	Presence or absence of change in tooth color	Application site	Treatment
1	Presence	Maxillary and mandibular anterior teeth	Placebo gel without hydrogen peroxide - Formula e Açao – Sao Paulo/SP
			- without activating source
2	Presence	Maxillary and mandibular anterior teeth	6.0% hydrogen peroxide –White Class with Calcium, FGM – Joenville/SC
			- without activating source
3	Presence	Maxillary and mandibular anterior teeth	7.5% hydrogen peroxide –White Class with Calcium – FGM – Joenville/SC
			- without activating source
4	Presence	Maxillary and mandibular anterior teeth	7.5% hydrogen peroxide – Oral-B 3D White, Oral-B - EUA
			- without activating source

For each group, 16 individuals enrolled at the Dental Clinic of the University Nove de Julho will be selected. After receiving all necessary information regarding the study, including the procedures and possible risks, the volunteers who agree to participate and their respective guardians will sign a statement of informed consent.

### Tooth whitening method using 6.0% or 7.5% hydrogen peroxide gel

Castings will be made of the volunteers’ upper and lower arches and plaster will be poured into the molds. Individual plastic molds (vacuum formed, flexible acetate 1 mm in thickness) will be made for each arch using the plaster models. After fitting the molds in the oral cavity, the volunteers will receive oral and written instructions on how to use the whitening gel and will be instructed to perform proper oral hygiene with dental floss and a toothbrush prior to the application of the whitening agent. The volunteers will be instructed to use the molds with whitening gel (6.0% or 7.5%, depending on which group the volunteer is in) one hour a day for 7 days.

### Tooth whitening method using placebo gel without hydrogen peroxide

Castings will be made of the volunteers’ upper and lower arches and plaster will be poured into the molds. Individual plastic molds (vacuum formed, flexible acetate 1 mm in thickness) will be made for each arch using the plaster models. After fitting the molds in the oral cavity, the volunteers will receive oral and written instructions on how to use the whitening gel and will be instructed to perform proper oral hygiene with dental floss and a toothbrush prior to the application of the whitening agent. The volunteers will be instructed to use the molds with whitening gel one hour a day for 7 days. Then 30 days after the beginning of treatment, the volunteers in this group will receive the whitening treatment with 6.0% hydrogen peroxide gel, following the same instruction as the first gel used.

### Tooth whitening using whitening strips

The participants in this group will receive four disposable whitening strips for daily individual use. The volunteers will be instructed to perform proper oral hygiene with dental floss and a toothbrush prior to the application of the whitening strips. The volunteers will receive oral and written instructions on how to use the whitening strips, which will be placed on the surface of the teeth for 30 minutes twice a day for 7 days.

### Evaluations

Evaluations of dental sensitivity, discoloration and satisfaction with the whitening will be carried out for all groups at the following times: pre-treatment period (T1), immediately following the first treatment period (T2), after 7 days (T3), after 30 days (T4), after 6 months (T5), after 12 months (T6), after 24 months (T7) and after 48 months (T8).

### Assessment of dental sensitivity

The volunteers will be asked to assess the degree of sensitivity at all evaluation times, using a visual analog scale. This is a numeric scale that runs from 0 (absence of sensitivity) to 10 (maximum sensitivity) [14].

### Colorimetric evaluation

The tooth color of all participants will be evaluated before and after the whitening procedures. Color readings of the vestibular region of the maxillary and mandibular anterior teeth will be performed by a previously trained examiner blinded to the allocation of the volunteers to the different groups. A digital spectrophotometer will be used for the colorimetric evaluation.

### Calculation of sample size

Considering a difference of 2.8 in *W*, 95% power, 5% error and standard deviation of 2.1 based on data from the literature [15], a minimum of 16 participants are needed for each group (see Table 1), to which 15% will be added to compensate for possible losses, totaling 19 volunteers per group. This calculation was made using the STATISTICA program.

### Organization and statistical treatment of data

The data will be tabulated and treated with the aid of SPSS 12.0 for Windows. Descriptive analysis of the data will be performed. Either the chi-squared test or Fisher’s exact test will be used to determine associations among the categorical variables. Student’s *t*-test and analysis of variance (ANOVA) will be used to compare mean colorimetric data. Pearson’s correlation coefficients will be calculated to determine the strength of correlations among the continuous variables. The level of significance will be set at 95% (*P* <0.05).

## Discussion

Tooth whitening can be performed in a dentist’s office or with the use of a home kit [3]. At-home whitening should be supervised by a dental professional and consists of the application of different concentrations of a peroxide gel (carbamide or hydrogen) to the teeth with the aid of personalized molds [9]. A number of publications have attested to the efficacy and biological safety of the at-home method [2, 10, 16]. Since the first proposal for home tooth whitening by Haywood and Heymann [9], both *in vitro* and *in situ* studies have demonstrated that whitening does not harm dental tissues and allows surprising esthetic results when properly employed [1, 17].

It is important to understand the action mechanism of the whitening agent and possible chemical interactions with dental tissues to minimize undesirable effects, such as tooth sensitivity. Hydrogen peroxide (H_2_O_2_) is the main whitening agent employed. It is a thermally unstable free radical with a low molecular weight, which penetrates the enamel and dentin through diffusion [18]. Complex molecules of organic pigments in the tissues are broken down into simpler hydrophilic molecules through an oxidation-reduction reaction by the action of perhydroxyl ions originating from the degradation of H_2_O_2_ [1]. These simpler molecules are easily removed from the dental tissue when in contact with water, thereby providing the desired whitening effect [5, 6, 16–20]. This is the action mechanism of peroxide in both the whitening process performed in the dentist’s office and the self-administered method performed in the comfort of the patient’s own home [1, 5, 6].

The concentration of the whitening agent and exposure time on the dental surfaces are important aspects to consider. Haywood and Heymann [9] proposed the home whitening of vital teeth with a 10% carbamide peroxide gel applied with the aid of molds. This protocol is still considered the gold standard for comparisons with novel methods. The main problems reported in studies involving different concentrations of carbamide peroxide (10 to 22%) are linked to the duration of use and contact between the gel and dental surfaces (an average of 6 hours, which is considered high by current standards) [1, 6, 19, 20] and the occurrence of gingival irritation and dental sensitivity during treatment [3].

Currently, carbamide peroxide has been replaced by hydrogen peroxide in molds at concentrations of 6 to 9.5%. With the aim of accelerating the home whitening process, H_2_O_2_ has become increasingly popular. The main advantage is the shorter application time (30 to 90 minutes), through the greater concentration of the product (10% carbamide peroxide leads to the formation of 3.5% H_2_O_2_) [18].

Most authors agree that whitening agents greatly improve a patient’s self-esteem [4-6,21]. The benefits of current whitening systems and the desires of consumers, fueled to some extent by the standards of beauty dictated by society [1], have stimulated the development of over-the-counter products sold in pharmacies and supermarkets without the need for a prescription or professional supervision [1, 10, 11]. These whitening products first appeared in the USA in 2000 as an alternative to dental staining treatment, with a lower cost in comparison to traditional treatment by dentists [10]. In the last 10 years, other over-the-counter whitening agents have been added, such as toothpastes, mouthwashes and chewing gum [10].

Despite the increase in over-the-counter whitening products, the small concentrations of peroxide lead one to have doubts regarding their true whitening potential [1, 11]. Moreover, the fact that these materials are employed without technical supervision causes discomfort in the scientific community, especially with regard to the abrasiveness of the components, possible morphological alterations and adverse effects on enamel exposed to more concentrated whitening agents [1, 10–13, 17]. Whitening strips and varnishes containing peroxide fall into this category of freely commercialized products without professional supervision, exposing individuals to allergic reactions, the ingestion of the whitening agent and dental sensitivity [12, 13]. Moreover, the lack of professional monitoring indicates a greater risk of the improper use of these products [11].

The safety, efficacy, long-term color stability and discomfort (dental sensitivity and gingival irritation) of over-the-counter whitening products need to be evaluated, quantified and described in the scientific literature.

## Trial status

The proposed study is currently in the recruitment phase and evaluation of tooth color.
